# Aswan Heart Centre: Fundamental values

**DOI:** 10.21542/gcsp.2024.1

**Published:** 2024-01-03

**Authors:** Ingi Farid

**Affiliations:** Human Resources Director, Magdi Yacoub Heart Foundation, Cairo, Egypt

To celebrate Aswan Heart Centre’s 15th Anniversary, which is an integral and core part of the Magdi Yacoub Foundation, it was decided to review the Values that have contributed significantly to the progress of AHC. To do this, we had a consensus meeting and the proceedings of the meeting represented our core values.


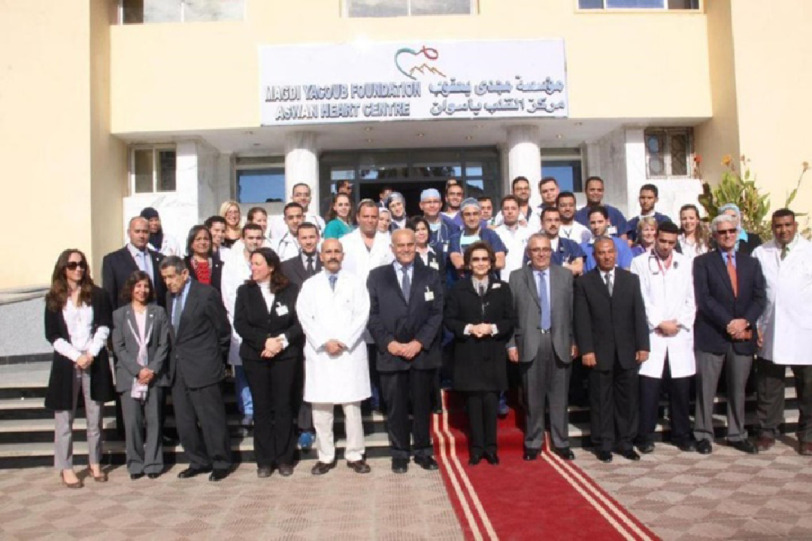


Reintroducing and reiterating our Values is an important step in ensuring the sustainability of the guiding principles and beliefs of our Foundation.

The Values represent the moral compass that shapes the culture, behavior, and decision-making processes. By embedding these Values, we are maintaining the legacy of AHC for all future generations.


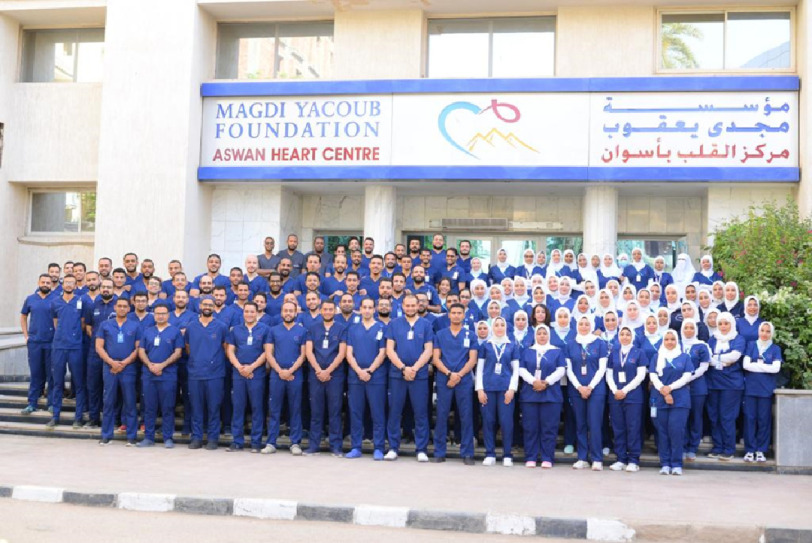


The Values were thoroughly chosen from a wide array of values and concepts and then the core Values were voted for by all Aswan Heart Centre employees.

*Our core values are CROPS. The acronym for our Values stems from the idea of growth and providing resources.*

• **C****ommitment & Transparency:** We are dedicated to our mission and the well-being of our patients and community. We demonstrate unwavering dedication, reliability, and accountability in everything we do. Our commitment extends to providing continuous learning and development opportunities for our team members. We are self-disciplined, resilient and persistent. We overcome obstacles to fulfill our obligations to others and to the workplace. We adhere to our goals no matter what the circumstances are. We conduct our mission with clarity and transparency.
• **R****espect:** We treat every individual with dignity, empathy, and fairness. We embrace our diversity and value the unique perspectives and contributions of each person, fostering a friendly environment for all.   We value everyone and treat all employees, clients and patients in an egalitarian fashion.
• **O****ne Team**: We believe in the power of collaboration and unity. We work together as a cohesive team, leveraging each team member’s strengths and expertise to deliver exceptional care and support to our patients. We value open communication, cooperation, and respect among all team members. We have a sense of belonging and are proud to be part of the Foundation. We are a team that celebrates our collective success.
• **P****atient First:** Our Foundation’s top priority is always the well-being and care of our patients. We are committed to providing the highest quality of healthcare services, ensuring their safety, comfort, and satisfaction. We are compassionate, kind and caring to everyone we encounter. We work cohesively and collaboratively. We put our patients at the heart of everything we do. We are Patient Centric and help people with kindness and empathy.
• **S****erving with Heart:** We approach our work with compassion, empathy, and kindness. We genuinely care about the needs of our patients, their families, and our colleagues and community. We go above and beyond to ensure their comfort, support, and overall well-being and are excited and passionate about shared objectives.

These core values will embody the principles that guide our Foundation moving forward and shape our culture, and drive our actions. By upholding these values, we strive to deliver exceptional healthcare services and make a positive impact on the lives of those we serve both locally and globally.


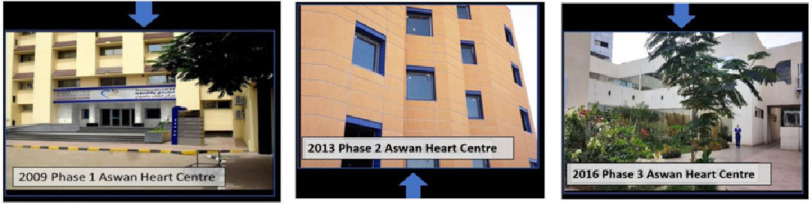



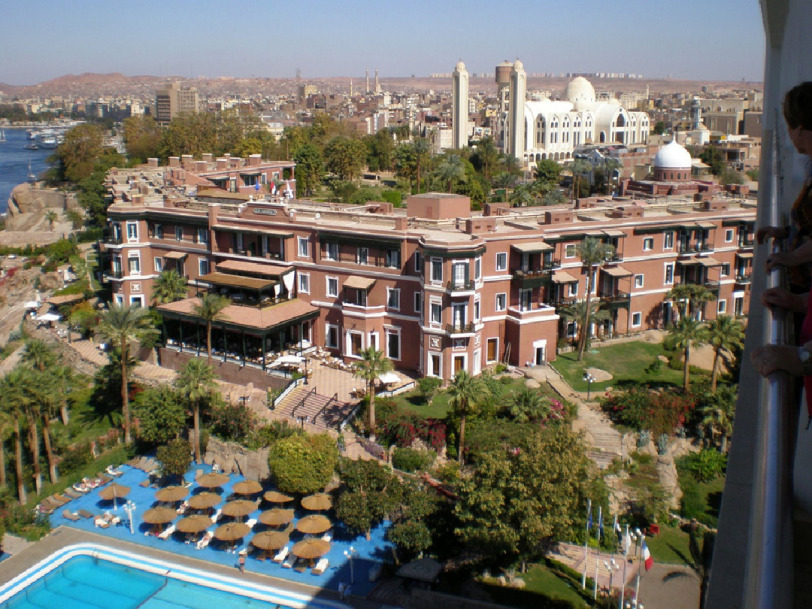



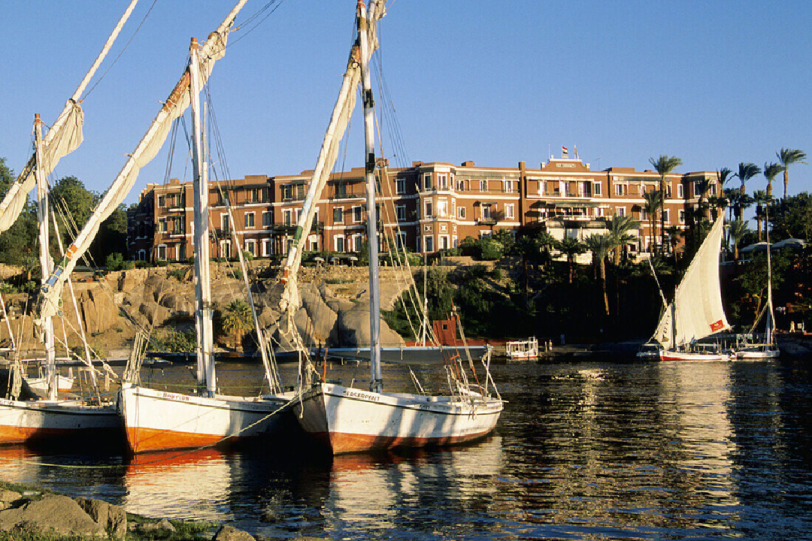


## Acknowledgement

I would like to thank all the employees of Aswan Heart Centre for their contribution and articulation of the Values, especially the Values Committee and the Human Resources Team whose names are listed below:

Ahmed ElDowaik

Ahmed Nabil

Dina ElGazzar

Doaa ElGhoneimy

Emad Nasr

Hatem Hosny

Ibrahim Farrag

Karam Mahmoud

Mahmoud Abdelhay

Mahmoud Nagdy

Mai ElRihany

Marwan Afifi

Mohamed Zakaria

Perihan Khairat

Ramy Adel

Tia Shehata

Yasmine Aguib

Zeina Tawakol

